# Changes in Spatiotemporal Patterns of 20th Century Spruce Budworm Outbreaks in Eastern Canadian Boreal Forests

**DOI:** 10.3389/fpls.2018.01905

**Published:** 2018-12-21

**Authors:** Lionel Navarro, Hubert Morin, Yves Bergeron, Miguel Montoro Girona

**Affiliations:** ^1^Département des Sciences Fondamentales, Université du Québec à Chicoutimi, Saguenay, QC, Canada; ^2^Chaire Industrielle CRSNG-UQAT-UQAM En Aménagement Forestier Durable, Institut de Recherche sur les Forêts, Université du Québec en Abitibi-Témiscamingue, Rouyn-Noranda, QC, Canada; ^3^Ecology Restoration Group, Department of Wildlife, Fish and Environmental Studies, Swedish University of Agricultural Sciences (SLU), Umeå, Sweden

**Keywords:** black spruce, climate change, dendroecology, GIS, insect outbreaks, landscape ecology, natural disturbances, sustainable forest management

## Abstract

In scenarios of future climate change, there is a projectedincrease in the occurrence and severity of natural disturbances inboreal forests. Spruce budworm (*Choristoneura fumiferana)*(SBW) is the main defoliator of conifer trees in the North American boreal forests affecting large areas and causing marked losses of timber supplies. However, the impact and the spatiotemporal patterns of SBW dynamics at the landscape scale over the last century remain poorly known. This is particularly true for northern regions dominated by spruce species. The main goal of this study is to reconstruct SBW outbreaks during the 20th century at the landscape scale and to evaluate changes in the associated spatiotemporal patterns in terms of distribution area, frequency, and severity. We rely on a dendroecological approach from sites within the eastern Canadian boreal forest and draw from a large dataset of almost 4,000 trees across a study area of nearly 800,000 km^2^. Interpolation and analyses of hotspots determined reductions in tree growth related to insect outbreak periods and identified the spatiotemporal patterns of SBW activity over the last century. The use of an Ordinary Least Squares model including regional temperature and precipitation anomalies allows us to assess the impact of climate variables on growth reductions and to compensate for the lack of non-host trees in northern regions. We identified three insect outbreaks having different spatiotemporal patterns, duration, and severity. The first (1905–1930) affected up to 40% of the studied trees, initially synchronizing from local infestations and then migrating to northern stands. The second outbreak (1935–1965) was the longest and the least severe with only up to 30% of trees affected by SBW activity. The third event (1968–1988) was the shortest, yet it was also the most severe and extensive, affecting nearly up to 50% of trees and 70% of the study area. This most recent event was identified for the first time at the limit of the commercial forest illustrating a northward shift of the SBW distribution area during the 20th century. Overall, this research confirms that insect outbreaks are a complex and dynamic ecological phenomena, which makes the understanding of natural disturbance cycles at multiple scales a major priority especially in the context of future regional climate change.

## Introduction

The boreal forest is the second-largest terrestrial biome in the world, covering 14 million km^2^. It forms a circumpolar forest belt (Burton et al., 2003) that represents about 25% of the world’s forests (Dunn et al., 2007). At present, two thirds of this surface is managed for wood production, and this proportion accounts for 37% of the global wood supply (Gauthier et al., 2015). However, an increasing number of studies predict marked consequences of climate change on boreal ecosystems through modifying the dynamics of natural disturbances at different scales and increasing the frequency and severity of events such as wildfires or insect outbreaks (Overpeck et al., 1990; Dale et al., 2001; Millar et al., 2007; Seidl et al., 2014, 2017; Alifa et al., 2017). Thus, improving our understanding of the variability of natural disturbance cycles at multiple scales will be a major challenge in the mitigation and adaptation of boreal forests and their management to climate change.

Natural disturbance regimes determine the dynamics, structure, and composition of forests by altering ecosystem functioning (Anyomi et al., 2016; Montoro Girona et al., 2018b). Insect outbreaks are a key disturbance to consider in forestry planning due to the important economic and ecological implications of these events (Sturtevant et al., 2015). Insect outbreaks affect timber supplies and have a marked impact on overall forest productivity and dynamics. Among all the major insect pests, spruce budworm [*Choristoneura fumiferana* (Clemens)] (SBW) is the most important defoliator of conifer trees in North American boreal forests (Hardy et al., 1983; Morin and Laprise, 1990). In Canada, more than 90% of spruce and fir forests are affected cyclically by SBW outbreaks, and more than 50% of the annual loss of volume caused by insect damage is attributed to SBW-related defoliation (Natural Resources Canada, 1994). Whereas the consequences of defoliation remain relatively moderate in the western Canada, in the eastern portions of Canada, SBW is responsible for significant losses for the forest industry through high tree mortality and a loss of forest productivity (MacLean, 2016).

SBW outbreaks are complex phenomena influenced by multiple factors that include the affected tree species, the specific ecoregion, and regional climate conditions (MacLean, 2016). Although insect outbreaks play an important role in forest dynamics, most studies involving SBW focus on the relationship with its primary host, balsam fir [*Abies balsamea* (L.) Mill.]. Mortality occurs in fir stands after 4 years of severe defoliation and outbreaks affect a very high proportion of trees (MacLean, 1980; Bergeron et al., 1995). For secondary hosts, such as black spruce [*Picea mariana* (Mill.) BSP], the damage (and death) of tree tops and branches is often accompanied by reductions in growth of up to 75% (MacLean, 1984; Nealis and Régnière, 2004). In black spruce, the resistance to defoliation is the result of a phenological asynchrony between the insect and its host (Volney and Fleming, 2000; Pureswaran et al., 2015). As the buds of black spruce burst 14 days later than those of balsam fir, the former is protected from severe SBW-related damage (Nealis and Régnière, 2004) since SBW emergence is synchronized to balsam fir bud burst. Indeed, although SBW can reach high latitudes corresponding to the distribution area of balsam fir (Harvey, 1985; Payette, 1993; Levasseur, 2000), its impact on the black spruce domain is lower, especially in situations where a cold summer prevents eggs from hatching, disrupting the annual cycle of the insect (Nealis and Régnière, 2009). Thus, it is expected that epidemic cycles should be more difficult to identify in the spruce–moss domain, the ecoregion that supports most of the timber industry in eastern Canada given its extent and the excellent wood properties of black spruce (Saucier, 1998; Zhang and Koubaa, 2008).

The reconstruction of insect outbreak cycles at the landscape scale is a major challenge as aerial surveys of defoliation – conducted annually since the 1960s – cover only one outbreak in the last century and are concentrated mostly in the balsam fir area. Dendroecological approaches are a reliable alternative for studying natural and anthropic disturbances in forest ecosystems at a fine resolution (Boulanger et al., 2012; Montoro Girona et al., 2016, 2017). Tree rings provide indirect measurements of insect activity, through the identification of years of growth reduction related to insect outbreaks, thereby allowing the reconstruction of SBW cycles at multiple scales (Morin and Laprise, 1990; Morin et al., 1993; Krause, 1997; Jardon, 2001; Boulanger and Arseneault, 2004; Boulanger et al., 2012). In regard to the spatial extent of SBW outbreaks, some studies have attempted to produce a portrait of past events via both modeling from aerial survey datasets (Fleming and Candau, 1998; Gray and Mackinnon, 2006) or tree-ring analysis (Jardon, 2001). However, the spatiotemporal changes of outbreak dynamics at the landscape scale over the last century in North American boreal forests remains poorly known.

The main goal of this study is to reconstruct the SBW outbreaks during the 20th century at the landscape scale and to evaluate changes in the spatiotemporal patterns in terms of distribution area and severity. For this, we use dendroecological data collected from the eastern Canadian boreal forest. We hypothesize that the spatial pattern will be similar from one outbreak to another with some variation in terms of intensity and expansion. We expect the last outbreak of the 20th century to have a greater expansion in the spruce domain. This would give credence to the hypothesis of a northward shift in the distribution of SBW in Quebec over the last cycles. However, we expect to observe a time lag in the emergence of the epidemic in the north, as well as a lag in growth reductions; both lags occur due to the lower susceptibility of black spruce stands. To improve the understanding of spatial temporal patterns of SBW activity, historical climatic data were used to examine the influence of precipitation and temperature on outbreaks periods.

## Materials and Methods

### Study Area

**FIGURE 1:**
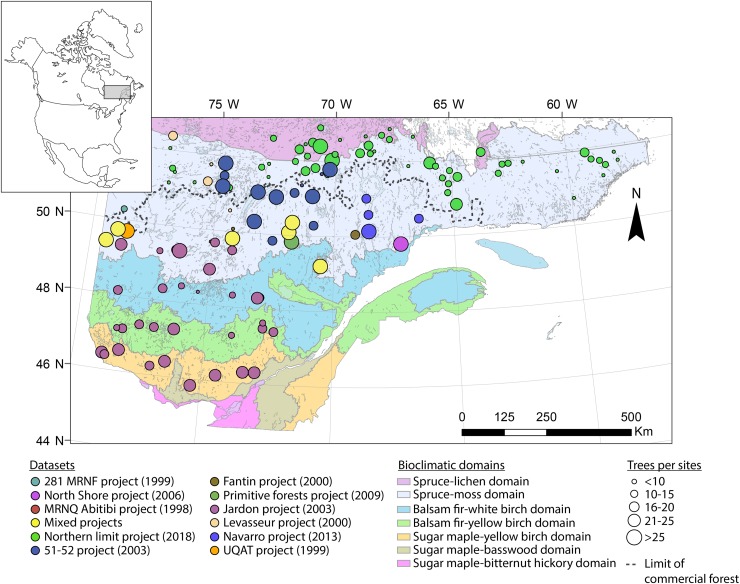
Location of study sites in Quebec (Canada). The different colors correspond to the various original datasets.

The study area is located in the boreal zone of Quebec (Canada) and lies within an area 45.5–53°N and 58–79°W, thereby covering nearly 800,000 km^2^ (Figure 1). This research involves a gradient of stand structures and ecoregions from closed, dense forests in the fir and spruce–moss domain to the south, to the more open and fragmented forests in the spruce–lichen domain to the north. The study area crosses the northern limit of the commercial forest, separating managed forests to the south from unmanaged ones to the north. The coniferous forest landscape is dominated by pure black spruce stands in the north and mixed forests of white spruce, fir, and broad-leaved trees in the south. Regional climate is subpolar humid with a growing season of ≥170 days in the fir domain to a cold subpolar subhumid climate having a much shorter growing season (≤100 days) in the spruce domain (Rossi et al., 2011). The eastern portion of the study area has a greater annual precipitation (950–1350 mm), and the fire return interval is longer in this portion at 270 to >500 years (Cyr et al., 2007).

### Data Compilation and Experimental Design

We undertook a data collection strategy to obtain the maximum amount of dendroecological data available for the years 1900–1990 from the study area. This database incorporated sites from eight projects undertaken at the University of Quebec in Chicoutimi over the last 20 years, two datasets from the Canadian Forest Service, and one project from the University of Quebec in Abitibi-Témiscamingue (Table 1). The dataset was complemented by an important field survey by Natural Resources Canada, undertaken between 2005 and 2010 in the northern portion of the study area (northern limit project). In this survey, more than 800 sampling plots (400 m^2^ each) were sampled. In each plot, wood disks were collected from seven dominant living trees and three dominant living saplings.

**Table 1:** Datasets compiled for this study.

Dataset name	Sampling date	Publication	Species	Tree samples	Max age	Sites Nbr
*281 MRNF*	1999	–	*P. mariana*	15	251	1
*Project North shore*	2006	–	*P. mariana*	69	234	1
*MRNQ Abitibi*	1998	–	*P. mariana*	9	205	1
*Mixed projects*	–	–	*P. mariana*	962	309	6
*Project Northern limit*	2018	Present publication	*P. mariana*	738	346	56
*Project 51–52*	2011	Tremblay et al., 2011	*P. mariana*	992	292	13
*Project Fantin*	2000	–	*P. mariana*	40	179	3
*Primitive forests*	2009	–	*P. mariana*	50	275	1
*Project Jardon*	2003	Jardon et al., 2003	*P. glauca*	608	250	32
*Project Levasseur*	2000	Levasseur, 2000	*P. mariana*	203	253	4
*Project Navarro*	2013	Navarro, 2013	*P. mariana*	120	291	4
*Project UQAT*	1999	–	*P. mariana*	31	176	1
Total				3837		123


Due to the size of our study area and the diversity of datasets sources, the original dataset was filtered to delete sites having a low number of samples (<8) and to keep only black spruce having an age of >100 years. The age criteria was established to guaranty that tree samples were able to register multiple insect outbreaks during the 20th century providing long chronologies across the study area. Based on the scale of this metanalysis and to maximize the number of trees per location, we aggregated some sites from the same ecoregions if they were close enough (≤20 km). This dataset is original and is valuable due to the size of the study area, the high number of trees used in this study, and the inclusion of new chronologies at the limit of the commercial forest in remote areas that are not accessible by road (Table 1 and Figure 1).

### Dendroecological Data

We selected trees based on dominant species criteria to ensure that the samples were representative of the study stands. All sites were composed exclusively of black spruce, with the exception of Jardon et al. (2003) where the samples were composed of white spruce (Table 1). For this study, a total of 3837 samples were used. The samples were prepared, measured, and analyzed based on standard dendroecological protocols (Krause and Morin, 1995). Breast height collected wood disks were air-dried and sanded before tree rings were measured with a WinDendro^TM^ system (Guay et al., 1992) or a manual Henson micrometer having an accuracy of 0.01 mm. Measurements of tree rings covered the entire life of the sampled tree, and the ring patterns were cross-dated using COFECHA (Holmes, 1983). We applied a double detrending method having a 50-year window spline and a negative exponential using ARSTAN (Holmes et al., 1986). Detrending reduced the effects of tree age, genetic growth potential, microsite and stand history, as well as minimizing the effect of climate allowing trees of different growth rates to be compared (Fritts, 1971). Autocorrelation in standardized time series was not removed for the sake of uniformity with similar studies (e.g., Krause, 1997; Boulanger and Arseneault, 2004; Tremblay et al., 2011; Boulanger et al., 2012). The detrended chronologies were averaged to produce a mean standardized chronology for each stand.

For the purpose of extracting the climatic signals in host series, most studies use a host–non-host correcting method using the OUTBREAK program (Holmes and Swetnam, 1996). In our case the lack of non-host species in northern latitudes (black spruce domain) make their use at large scales challenging. In order to overcome this issue, we used modeled climatic data (*see below*). An epidemic period was defined as a growth reduction (≥1.28 SD on the mean standardized chronologies) of at least five consecutive years allowing 1 year of growth release (Jardon et al., 2003). The severity of insect outbreaks was defined by the proportion of trees at each site that presented such a pattern of growth reduction. This dendroecological approach was used on previous research on black spruce stands (Tremblay et al., 2011; Boulanger et al., 2012, Rossi et al., 2018).

### Data Analysis

To establish the patterns of SBW activity, spatial data related to insect outbreaks were interpolated based on the percentage of trees affected using an inverse distance weighted interpolation (Childs, 2004) with GIS analysis techniques from the function “Spatial Statistic” extension of ArcGIS 10.3 (ESRI Inc., 2017). Only the epidemic years are shown. The complete chronology can be found in the Supplementary Material (Supplementary Figure S1).

### Cluster and Hotspot Analysis

To evaluate the spatial synchrony of insect outbreaks, hotspot and coldspot analyses estimated spatial clustering among the study sites affected or not affected by SBW outbreaks in eastern Canada based on the Getis-Ord local statistic using a fixed distance band estimated as:

(1) $$G_{i}^{*}=\frac{\sum_{j=1}^{n}w_{i,\;j}\;x_{j}-\bar{X}\left(\sum_{j=1}^{n}w_{i,\;j}\right)}{S\sqrt{\frac{n\sum_{j=1}^{n}w2_{i,\;j}-\left(\sum_{j=1}^{n}w_{i,j}\right)}{n-1}}}$$

(2) $$\bar{X}=\frac{n\sum_{j=1}^{n}x_{j}}{n}$$

(3) $$S=\sqrt{\frac{\sum_{j=1}^{n}x_{j}^{2}}{n}-{\left(\bar{X}\right)}^{2}}$$

where *x_j_* corresponds to the percentage of trees affected for site *j, w_i,j_* is the spatial weight between feature *i* and *j*, and *n* is equal to the total number of sites.

Hotspot/coldspot fields were recognized based on statistically significant levels (i.e., 0.1, 0.05, or 0.01); these fell into hotspots, where high values were intermingled with high values and coldspots where low values were intermingled with low values. Areas where high values were surrounded by lower values and where low values were surrounded by higher values were considered as non-significant clusters. To summarize the overloading of maps, these outputs were also presented using Hovmöller diagrams (Persson, 2017). This tool is effective for displaying large amounts of data. It is a technique that is used frequently in atmospheric sciences (Du and Rotunno, 2018). This diagram represents the longitude (or latitude) versus time with the value of the dataset represented through color or shading. *RasterVis* and *LevelPlot* R packages were used to plot Gi^∗^Z-scores and *p*-values.

### Climate Model

To improve the interpretation of patterns in the dendrochronological data, we used a climatic model provided by the Climatic Research Unit at the University of East Anglia (CRU TS 3.10). This model is based on an updated gridded climate dataset across the global land areas (excluding Antarctica). The data available for our study area was provided by the Canadian Historical Temperature Database (Vincent and Gullett, 1999). The dataset is composed of monthly precipitation and mean temperature observations on a 0.5-degree latitude/longitude grid over the entire 20th century. Anomalies (positive and negative mean deviations) were estimated using the mean values for each cell for the period with best coverage (1961–1990) (Jones et al., 2012). These anomalies were averaged for each season and used as explanatory variables to compute Ordinary Least Squares linear regression in order to model the relationship between climate variables and the percentage of affected trees (dependant variable). In the absence of non-host chronologies at the landscape scale, this procedure allows us to better assess the proportion of the variability relative to climatic factors versus the unexplained variation (residuals) which can be attributed to SBW outbreaks. Spatial autocorrelation (Moran’s Index) was conducted on standard deviations of the residuals to analyze its clustering level.

## Results

**FIGURE 2:**
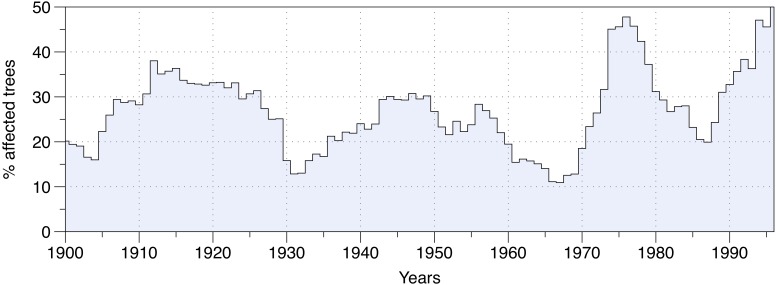
The proportion of trees affected by outbreak conditions in the study area.

The percentage of affected trees over the entire study area revealed three main SBW outbreak periods in eastern Canadian forests over the last century (Figure 2). Each insect outbreak differed in terms of duration and severity. The first outbreak occurred between 1905 and 1930, and nearly 40% of the studied trees were affected by SBW activity at the epidemic’s peak (1914). The second outbreak was the longest infestation, lasting from 1935 to 1965, although it had the lowest severity level with only 30% of trees being affected during the peak (around 1950). The third outbreak from 1968 to 1988, was the shortest, yet it was the most severe affecting nearly 50% of the studied trees in 1977 (Figure 2).

**FIGURE 3:**
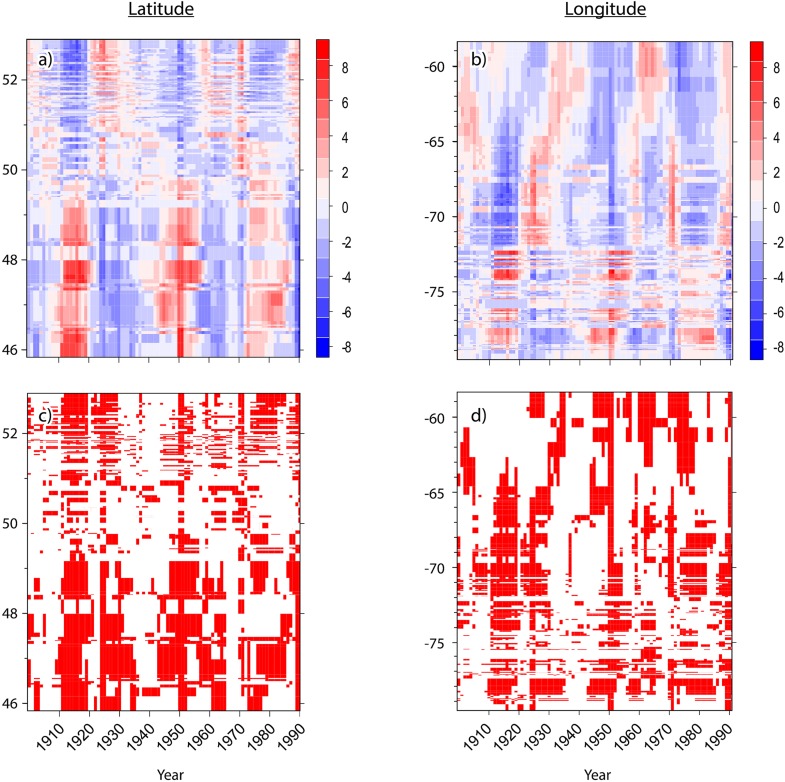
Hovmöller diagrams for the spatiotemporal patterns of spruce budworm outbreak impacts during the last century in eastern Canadian boreal forests where **(A,B)** represent the Z-scores of Getis-Ord Hotspot analysis by latitude (left) and by longitude (right), respectively. Red represents hotspots having a high percentage of affected trees. Blue represents cold spots having a low percentage of affected trees. **(C,D)** present, the significance by latitude and longitude, respectively. Red, *p*-value < 0.05; White, *p*-value > 0.05.

**FIGURE 4:**
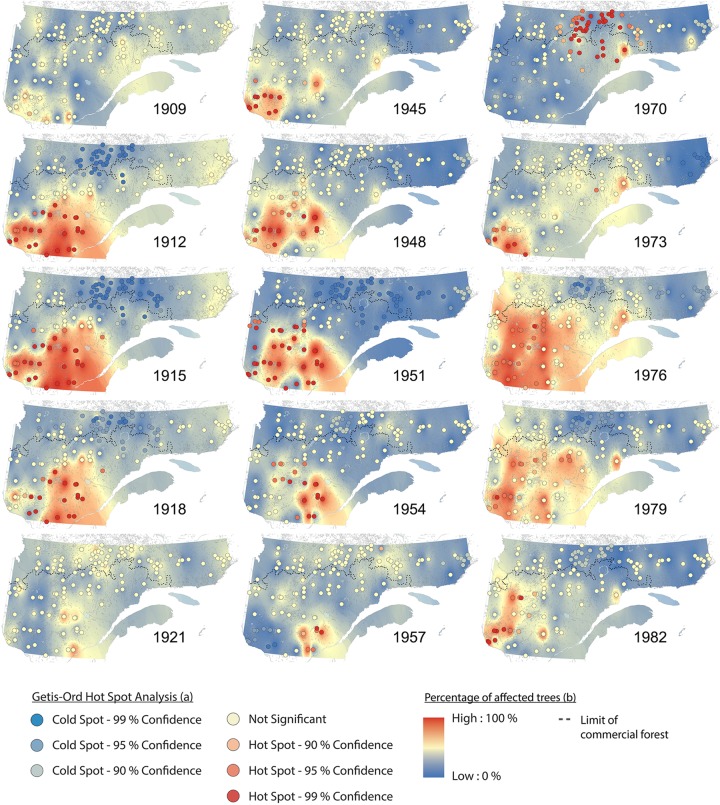
Spatiotemporal patterns of spruce budworm impacts (percentage of affected trees) and synchrony (Getis-Ord hotspot analysis) in eastern Canadian boreal forests for the three most important outbreaks over the 20th century.

Hotspot and cluster analyses revealed changes in the spatiotemporal patterns of SBW dynamics, as well as the impacts across the eastern Canadian boreal forest during these three periods of budworm outbreak over the last century (Figures 3, 4). From 1905 to 1910, no hotspots were recorded. This pattern indicates no synchronization at the landscape scale, although we detected a pattern of locally affected sites from the southwest to the east with a moderate percentage of affected trees (Figure 4 and Supplementary Figure S1a). A significant hotspot, composed of multiple sites was registered in the southwestern portions of the study area in 1911. This infestation reached a maximum affected area of ≈280,000 km^2^ in 1914, and then fell to a lower impact phase in 1921 (Figure 4 and Supplementary Figure S1b). This outbreak affected primarily white spruce sampled within the fir domain (Jardon, 2001). SBW activity demonstrated a temporal delay when the area north of the 50th parallel was affected at a later date (1920–1930), with a lower proportion of affected trees and a lower clustering level compared to previous events to the south of our study area (Figures 3, 4). A similar pattern was observed for the second insect outbreak; various sites recorded moderate to severe outbreaks at the local scale prior to the onset in 1944. Four spots had persistently high percentages of affected trees from the early 1940s (the Abitibi region, southwest of Lake Saint-Jean, the Upper North Shore, and Lake Mistassini). There was a cluster of sites in the southwestern region (1944) that formed a hotspot followed by an eastward expansion of moderate to severe SBW impact until 1957. This outbreak reached an affected area of ≈170,000 km^2^ in 1950 (Figure 4). The third outbreak had a widespread impact on stands of the fir domain from west to east across our study area, corresponding to 80% of the forest area (≈550,000 km^2^). This insect outbreak period was first recorded in the northern portion of the spruce–moss domain (especially in sites close to the limit of the commercial forest) in 1970. Analysis of the hotspots revealed a significant cluster of high values in the northern forest for a short period (1970–1971). A persistent pattern having a high percentage of affected trees was identified in the southwest in 1973, followed by an increasing eastward distribution until 1978, finally ending in a retraction to its original position from 1978 to 1982 (Figures 3, 4).

**FIGURE 5:**
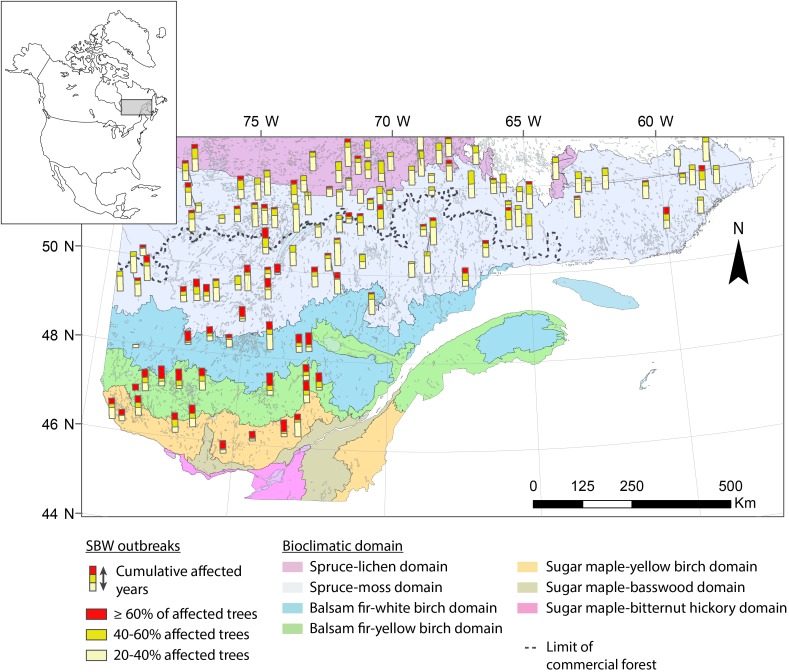
Cumulative years of infestation in the study area by category of severity.

Based on severity (number of affected trees) and duration (number of years), we determined the differences in SBW outbreak intensity between the northern and southern portions of the eastern Canadian forests over the last century (Figure 5). In the northern stands, tree-ring chronologies registered a higher number of years of weaker outbreaks than in the southern sites. However, severe SBW outbreaks were rare at the northern limit of the commercial forest during the 20th century (Figure 5). The southern portion of the study area was characterized by shorter, more severe, and more synchronized periods of SBW infestation.

**FIGURE 6:**
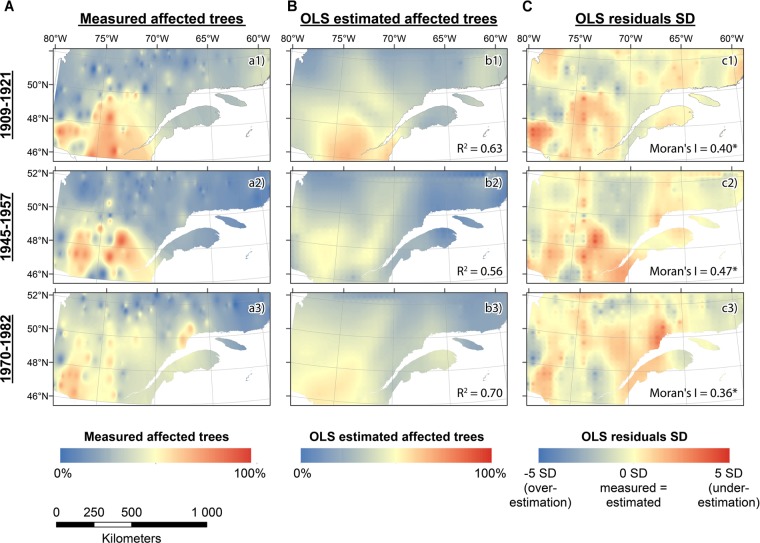
Ordinary Least Squares (OLS) model for each outbreak period. **(A)** Average measured percentage of affected trees over an outbreak period. **(B)** OLS estimated percentage of affected trees over an outbreak period using seasonal precipitation and temperature as explanatory variables according to the Table 2. **(C)** Residuals of the OLS model.

Climate regressions had relatively high *R^2^* values for each outbreak period, ranged from 0.56 to 0.70 (Table 2). However, the Koenker and the Jarque-Bera statistics indicated non-stationarity and heteroscedasticity. Thus, the relationship between climate and outbreak periods was spatially inconsistent and changed with explanatory variable magnitudes. Indeed, even if each explanatory variable was significantly correlated to the frequency of affected trees, and even if the Variance Inflation Factors (VIF) indicated no redundancy among explanatory variables (<7.5), the model was improperly specified. The Moran’s Index between 0.36 and 0.47 indicated significant tive clustering of the regression residuals. Thus, a key variable was missing and the model was mis specified suggesting that a key variable is missing and that the model is misspecified. Therefore, As a matter of fact, the spatial patterns of the regression residuals was is similar to the one of the measured percentage of trees affected and the higher values are consistently underestimated, in the southwest and southeast portion of the study area for the first and the second outbreak periods (Figures 6c1,c2) and in the southwest and the northeast for the third one (Figure 6c3).

**Table 2:** Ordinary Least Squares model for each outbreak period.

First outbreak: 1909–1921					
***R^2^***	**F-value**	**Koenker**	**Jarque-Bera**	**Moran’s I**	

**0.63**	**261.81**	**89.63**	**29.67**	**0.40**	

***Variable***	**Coefficient**	**SE**	**t-Statistic**	***P***	**VIF**

*Intercept*	-3.07	2.54	-1.21	0.2270	–
*Summer temperature*	45.36	2.3	19.73	**<0.0001**	2.93
*Autumn temperature*	-64.86	3.57	-18.12	**<0.0001**	3.21
*Winter temperature*	-12.39	2.32	-5.34	**<0.0001**	1.57
*Spring precipitation*	-1.09	0.1	-10.73	**<0.0001**	1.31

**Second outbreak: 1945–1957**					

***R^2^***	**F-value**	**Koenker**	**Jarque-Bera**	**Moran’s I**	

**0.56**	**132.65**	**93.60**	**26.95**	**0.47**	

**Variable**	**Coefficient**	**SE**	**t-Statistic**	***P***	**VIF**

Intercept	-3.73	2.17	-1.72	0.0864	–
Winter temperature	-35.62	2.16	-16.46	**<0.0001**	1.14
Autumn temperature	39.73	2.28	17.46	**<0.0001**	1.48
Spring temperature	22.67	3.86	5.87	**<0.0001**	1.18
Winter precipitation	0.71	0.1	7.34	**<0.0001**	1.44
Summer precipitation	-1.44	0.08	-17.52	**<0.0001**	1.25
Spring precipitation	0.35	0.1	3.5	**0.0005**	1.11

**Third outbreak: 1970–1982**					

***R^2^***	**F-value**	**Koenker**	**Jarque-Bera**	**Moran’s I**	

**0.70**	**247.79**	**34.84**	**33.40**	**0.36**	

**Variable**	**Coefficient**	**SE**	**t-Statistic**	***P***	**VIF**

Intercept	37.59	1.32	28.4	**<0.0001**	–
Winter precipitation	-1.18	0.16	-7.4	**<0.0001**	3.6
Autumn precipitation	-0.89	0.17	-5.12	**<0.0001**	1.6
Spring precipitation	1.4	0.14	9.8	**<0.0001**	2.66
Winter temperature	-50.27	2.94	-17.09	**<0.0001**	2.11
Autumn temperature	42	5.47	7.68	**<0.0001**	2.96
Spring temperature	39.67	8.13	4.88	**<0.0001**	2.13


*The variables included were selected among spring, summer, autumn and winter precipitation and temperature in order to best fit the model (Variance Inflation Factor-VIF < 7.5 and significant coefficient). Values in bold correspond to significant results (*P* < 0.05), SE correspond to standard error.*

## Discussion

Under most climate change scenarios, disturbance regimes are likely to be most pronounced within the boreal biome (Seidl et al., 2017). As a consequence, much research has been aimed on improving our understanding of fire (Cyr et al., 2016; Drobyshev et al., 2017; Portier et al., 2018), insect outbreaks (Boulanger et al., 2016; Boulanger et al., 2017b; Montoro Girona et al., 2018b), and windthrow (Anyomi et al., 2017; Saad et al., 2017) in the North American boreal forest. In the recent years, the eastern Canadian boreal forest has been experiencing a SBW outbreak, during which some of the most productive forest areas have been severely damaged (e.g., along the North Shore region), thereby with implications at the ecological (forest dynamics) and economic level (financial losses) due to the large extent of affected forest. As the frequency and severity of disturbances are expected to increase under future climate change scenarios, understanding the impact of SBW outbreaks in the past becomes essential for adapting to the uncertainties of climate change. In this study, we provide for the first time a landscape reconstruction of the spatiotemporal pattern of SBW dynamics over the last century across a vast study area of almost 1 million km^2^ in the eastern Canadian boreal forest. We also reveal the first evidence of the presence of endemic populations of SBW north of the 50th parallel.

This study demonstrated that SBW outbreaks have a major impact on forest ecosystems in terms of growth reduction that influences tree survival, regeneration, and succession (MacLean, 2016). Dendroecological series across the entire study area have identified three main periods of elevated SBW activity (Morin and Laprise, 1990; Jardon et al., 2003; Boulanger et al., 2012). Contrarily to our preliminary expectation, the first hypotheses was rejected, because each insect outbreak was manifested by a different spatiotemporal pattern, severity, and duration, thereby demonstrating the complexity of this ecological phenomenon.

### Spatiotemporal Patterns at the Landscape Scale

These different patterns manifest themselves by an expansion of the spatial extent of the affected area over the 20th century. This dynamic is confirmed at a wider temporal scale as outbreaks in the 19th century were less synchronous and presented a lower diffusion rate (Jardon et al., 2003). This could be the result of a long-term forest transformation process. Baskerville (1975) described SBW as a super silviculturist, killing overstory trees and promoting the development of shade-tolerant species, such as balsam fir (Morin, 1994; Morin and Laprise, 1997). Even if the anthropogenic influences on SBW dynamics remain a matter of debate (MacLean, 2016), we know that fire suppression, clear cutting, and insecticide spraying tend to favor the development of fir, SBW’s most vulnerable host (MacLean, 1980). This forest transformation process, enhanced by the diminution of fire frequency since the end of the Little Ice Age (Drobyshev et al., 2017), could have a major role in explaining the onset of the first SBW outbreak in the early 20th century (Bergeron and Leduc, 1998; Jardon et al., 2003). Furthermore, this outbreak occurred during one of the driest decades of the last century “1910–1920” (Girardin et al., 2009), reducing host trees’ vigor, and favoring their susceptibility to subsequent stresses, such as fire or SBW outbreaks (Flower et al., 2014; Berdanier and Clark, 2016).

The full spatial extent of this outbreak is described here for the first time at the landscape level using dendrochronological data and including sites from both fir and spruce domain. From 1909 to 1921, this outbreak affected mainly the fir domain south of the 50th parallel. Several local infestations were recorded in the years 1905–1909, prior to the synchronized outbreak in the southwestern portion in 1911 (Supplementary Figure S1a). This synchronization at a regional and landscape scale could stem from favorable weather conditions (Moran effect) (Myers, 1998; Royama et al., 2005) combined with the exchange of eggs by moth dispersion (Williams and Liebhold, 2000). The northern part of the study area was later affected moderately and synchronously during the 1920s. A second infestation (1935–1965) was smaller in extent, had a milder impact in more northern latitudes. However, this event does present the same pattern than the first outbreak with local infestations occurring during a few years prior to a synchronized outbreak at a wider scale. Our data tend to confirm the theory of Blais (1983) and suggest that an outbreak following a stand-replacing epidemic event will have a lower impact due to the establishment of less vulnerable younger stands. Finally, the 1968–1988 epidemic was the largest, most synchronous, and best-documented SBW outbreak in eastern Canada (Morin and Laprise, 1990; Morin et al., 2007). Its dynamics in the spruce domain presented a very different pattern from the earlier outbreaks, and it appears to have reached almost all study sites. Furthermore, although spruce are less vulnerable to SBW than fir (MacLean, 1980), many spruce stands were significantly impacted by this outbreak.

### Factors Involved in Spatiotemporal Patterns at Multiples Scales

These differences in spatiotemporal patterns could be explained by many factors, one of the most important being climate. In fact, as climate influences SBW population dynamics, it also causes stress to host stands causing them to be more vulnerable to subsequent biotically induced disturbances (De Grandpré et al., 2018a). Greenbank (1956) demonstrated that hot and dry summers influenced the onset of the 1912 and 1949 SBW outbreaks in New Brunswick. Hot summer temperatures are required for the insect to complete its life cycle (Pureswaran et al., 2015). Drought can also increase host vulnerability by enhancing the carbohydrate content of leaves (Mattson and Haack, 1987). The northern portion of our study area is characterized by cold and short summers that, at present, prevent the establishment of endemic populations. Early frosts in the northern stands prevent eggs from hatching (Pureswaran et al., 2015). Thus, the outbreak impacts observed on trees from the more northern sites during the 1920s could be evidence for the arrival of immigrant populations from southwestern Quebec. Recently, the use of weather radar has allowed the identification of such mass exodus events (Boulanger et al., 2017a). However, the 1968–1988 outbreak provides a different picture as severe and synchronous local epidemics were recorded in the early phases (1970–1971) of the outbreak within the North Shore region before any growth reduction was recorded in the fir domain (1972) (Figure 4 and Supplementary Figure S1e). This phenomenon matches with the distribution of our regression analysis residuals, discarding the hypothesis of a climatic event influence as a unique factor of growth reduction (Figure 6). This could therefore be the first evidence of the presence of endemic populations of SBW north of the 50th parallel. In addition, as black spruce phenology and overall shoot length increase in response to experimental warming (Bronson et al., 2009), this could have reduced the phenological asynchrony between the insect and host, thereby making northern sites more suitable for infestation. These arguments give weight to the hypothesis of a northward shift in the extent of SBW outbreaks during the 20th century (Régnière et al., 2012; Pureswaran et al., 2015). Gray (2013) identified the North Shore region and Gaspé Peninsula as the areas having the highest increases in outbreak severity and duration; this agrees with our dataset from the North Shore and with the onset area of the current infestation. Unfortunately, our dataset did not contain sites from the Gaspé Peninsula.

SBW dynamics recorded at the regional scale differ between the northern and southern portions of the study area. In the spruce domain, we observed more cumulative years of growth reduction than what was observed in the southern regions (Figure 5). However, we have found only a few occurrences of severe SBW impacts on black spruce in comparison to the fir domain where white spruces were periodically (and severely) affected. First, black spruce is less vulnerable to SBW than white spruce or balsam fir. This is due to an asynchrony of approximately 14 days in budburst phenology causing high rates of mortality for the second instar larvae trying to feed on this host (Nealis and Régnière, 2004). Despite the fact that white spruce buds burst in a time frame more similar to balsam fir than black spruce, white spruce also produces more buds that develop and lignify faster (Nealis and Régnière, 2004). According to MacLean (2016), balsam fir and white spruce, being less resistant species, are more prone to secondary mortality agents, such as shoestring root rot, *Armillaria mellea* (Vahl ex Fries), which occurs in most defoliated trees. In addition, southern mixed forest benefit from a greater diversity of SBW natural enemies, which could also explain the lower frequency and shorter duration of outbreaks in this zone (Cappuccino et al., 1998; Campbell et al., 2008).

### Methodological and Forest Management Implications

Similarities were observed between our spatiotemporal patterns and the aerial surveys of defoliation in the area (Gray et al., 2000; Gray and Mackinnon, 2006). Although these surveys were not conducted specifically to measure SBW impacts on black spruce at these latitudes, they still show an important spread of the epidemic in 1974, especially in the spruce–moss domain. It is possible that dendrochronology detects epidemic thresholds earlier as it is more sensitive, and the technique is better suited to black spruce. Indeed, growth reductions can be observed for defoliation levels that are not detectable with aerial surveys, which are categorial (none, light, moderate, severe).

The dendroecological approach has shown its effectiveness in the study of past insect outbreak dynamics (Jardon et al., 2003; Morin et al., 2007; Boulanger et al., 2012). Given the large amount of dendrochronological data that has been published during the last decades, large-scale meta-analyses are increasingly important and can provide a complete portrait of historical SBW outbreaks, placing recent events into a larger spatiotemporal context, and completing the existing monitoring proxies. Therefore, to provide a more relevant understanding of the SBW dynamics, future sampling efforts should be more homogeneous with sampling sites evenly distributed and with uniform sampling methods. Furthermore, the inclusion of northern latitude sites demonstrated its potential for improving our understanding of past outbreak patterns, but also the methodological difficulties associated with the inclusion of a secondary host in the analysis. The inclusion of such chronologies will be challenging, nonetheless, given the difficulties in accessing sampling sites, finding old trees able to provide long chronologies, and, moreover, the difficulty of finding non-host trees to refine the epidemic signal. We recommend continuing the collection of more samples from northern latitude sites, considering new areas (e.g., Ontario and Gaspe peninsula), as well as adding other species affected by SBW (e.g., balsam fir). Getting more details across the historical distribution area of SBW will improve the resolution of the existing dendroecological database. Based on our results concerning the potential links between climate and SBW activity (Figure 6), we suggest that future research should be developed to better discriminate the interactions connecting climate anomalies, as a triggering or interrupting factor of growth reduction, to SBW and its hosts dynamics.

Natural disturbance regimes are an integral part of boreal forest ecosystems, and silvicultural methods are now attempting to emulate their impacts by adapting more appropriate harvesting treatments (Kuuluvainen and Grenfell, 2012; Montoro Girona et al., 2018a). Many studies have focused on the role, impact, and frequency of fire cycles on the management of boreal ecosystems (Bergeron et al., 2002; Kuuluvainen and Grenfell, 2012); however, the understanding of the role and impacts of insect outbreaks remains incomplete, in particular at a larger scale (De Grandpré et al., 2018b; Robert et al., 2018). Understanding SBW disturbance regimes at the landscape-scale and implementing effective management strategies requires to define outbreak dynamics in both time and space (Bouchard and Pothier, 2010). Currently, sylvicultural practices aim to imitate fire disturbances promoting large clearcuts (Hunter, 1993; National Forest Database [N.F.D], 2016. In order to reduce boreal forest vulnerability to SBW outbreaks, some authors proposed to adapt sylvicultural treatments and forest management promoting the harvesting of the most susceptible stands such as mature fir stands (MacLean, 1980, 1996; Sainte-Marie et al., 2014) and favor uneven-aged spruce and mixed stands through silvicultural practices such as partial cuttings (Bergeron et al., 2017). Integrative multiple-disturbance research is needed to better understand the climatic and ecological context of insect outbreaks and to identify the type of interactions that occur during these events; as such, adequate management strategies can be developed in accordance with the forest structure at regional and local scales.

## Conclusion

Natural disturbance regimes define forest ecosystems by influencing their structure, species composition, and functional processes. The evaluation of outbreak periods during the last century demonstrated that SBW is a major disturbance event in eastern Canada, affecting large surfaces and having an impact on forest ecosystem dynamics. Landscape-scale reconstruction of the spatiotemporal patterns of SBW outbreaks in eastern Canadian forests highlighted three outbreaks during the 20th century, each having different spatiotemporal patterns, duration, and severity. This study revealed the diversity and complexity of outbreak dynamics over time as well as the importance of meta-analyses for better understanding the SBW patterns at the landscape scale and evaluating the impacts on forest ecosystems. Furthermore, this study represents a major contribution to forest ecology providing valuable data from remote sites located at the limit of commercial forests.

Under climate change, natural disturbances regimes and species’ distributions are expected to be altered. Based on dendroecological approaches, we demonstrated evidence of SBW activity north of the 50th parallel, adding weight to the hypothesis of a northward shift in the extent to the outbreaks during the 20th century (Régnière et al., 2012; Pureswaran et al., 2015). Finally, improving our understanding of natural disturbance cycles at multiple scales should be a priority for assessing boreal forest adaptation and modification to future climate change.

## Author Contributions

LN, MG, HM, and YB conceptualized and investigated the study. LN performed the data curation and administered the project. LN and MG designed the methodology, wrote the original draft of the manuscript, and edited the manuscript. HM provided the resources. HM and MG supervised the study. LN, MG, HM, and YB reviewed the manuscript. HM and YB contributed to the funding.

## Conflict of Interest Statement

The authors declare that the research was conducted in the absence of any commercial or financial relationships that could be construed as a potential conflict of interest.
