# Supplementary material for: Changes in Spatiotemporal Patterns of 20th Century Spruce Budworm Outbreaks in Eastern Canadian Boreal Forests
Source: Front Plant Sci. 2018 Dec 21;9:1905. doi: 10.3389/fpls.2018.01905 (PMC6308396; doi:10.3389/fpls.2018.01905)

**Supplementary material**

**Figure S1a.** Spatiotemporal pattern of spruce budworm impacts (percentage of affected trees) and synchrony (Getis-Ord hotspot analysis) in eastern Canadian boreal forests for the period 1900–1914.


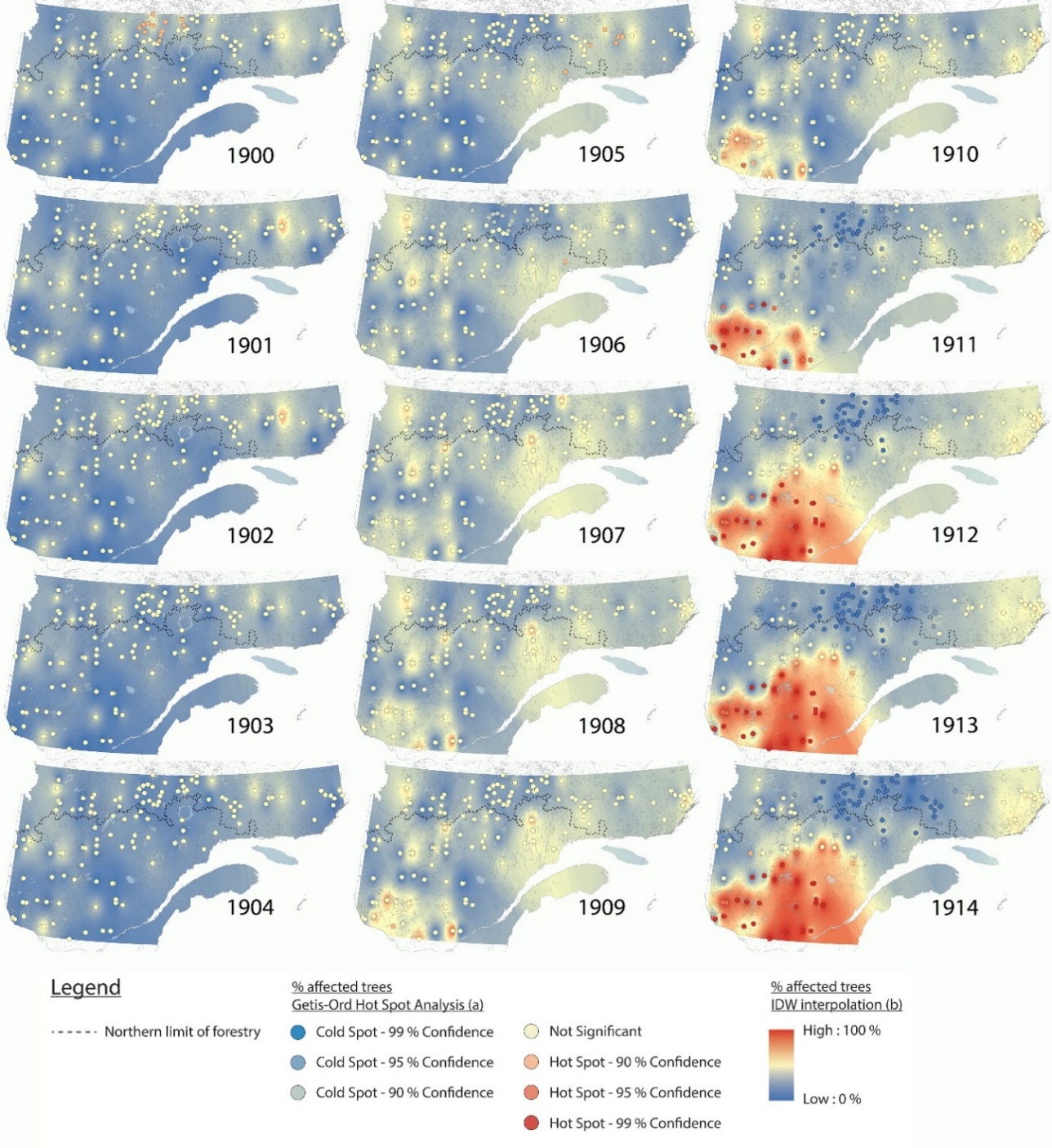


**Figure S1b.** Spatiotemporal pattern of spruce budworm impacts (percentage of affected trees) and synchrony (Getis-Ord hotspot analysis) in eastern Canadian boreal forests for the period 1915–1929.


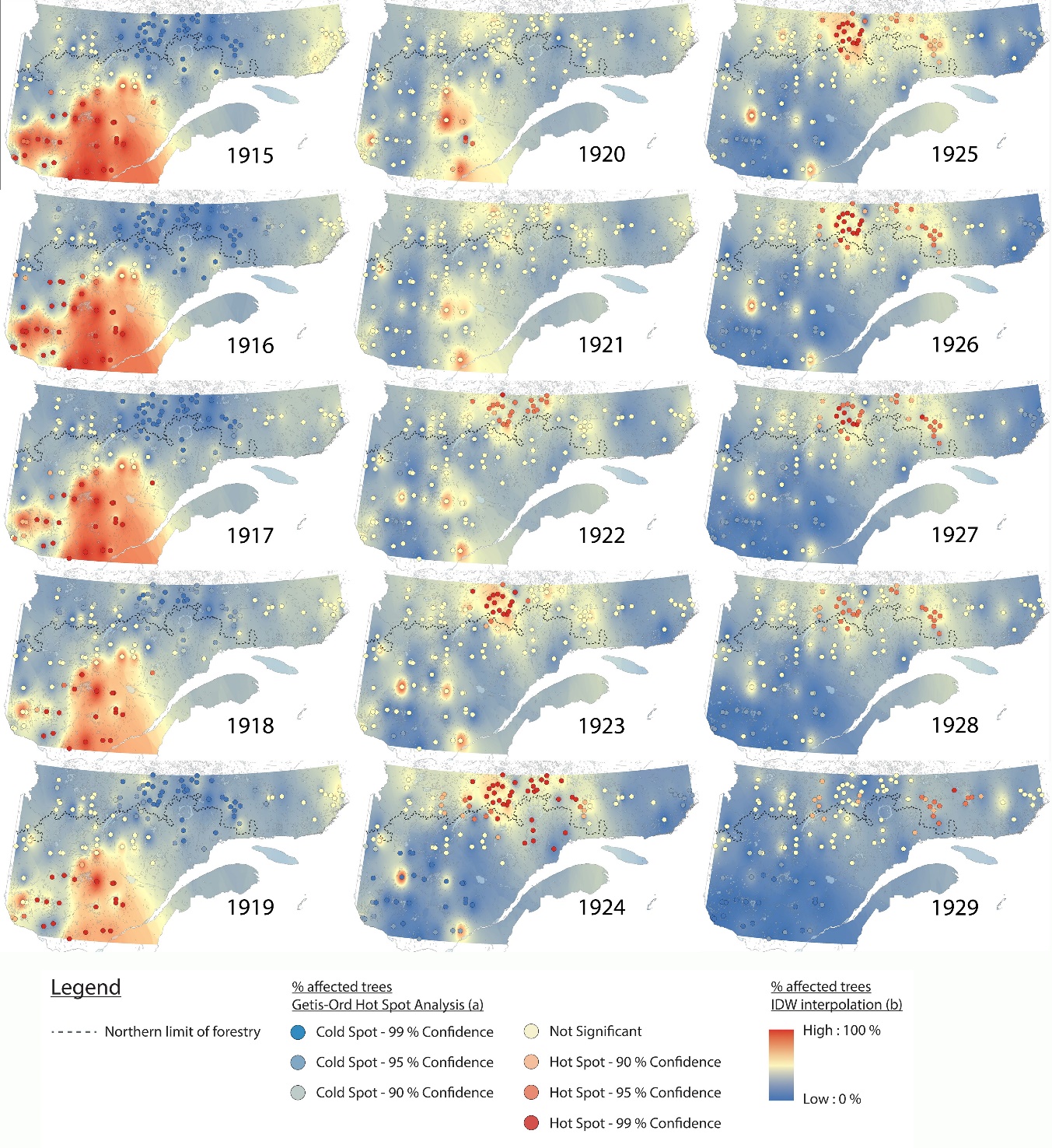


**Figure S1c.** Spatiotemporal pattern of spruce budworm impacts (percentage of affected trees) and synchrony (Getis-Ord hotspot analysis) in eastern Canadian boreal forests for the period 1930–1944.


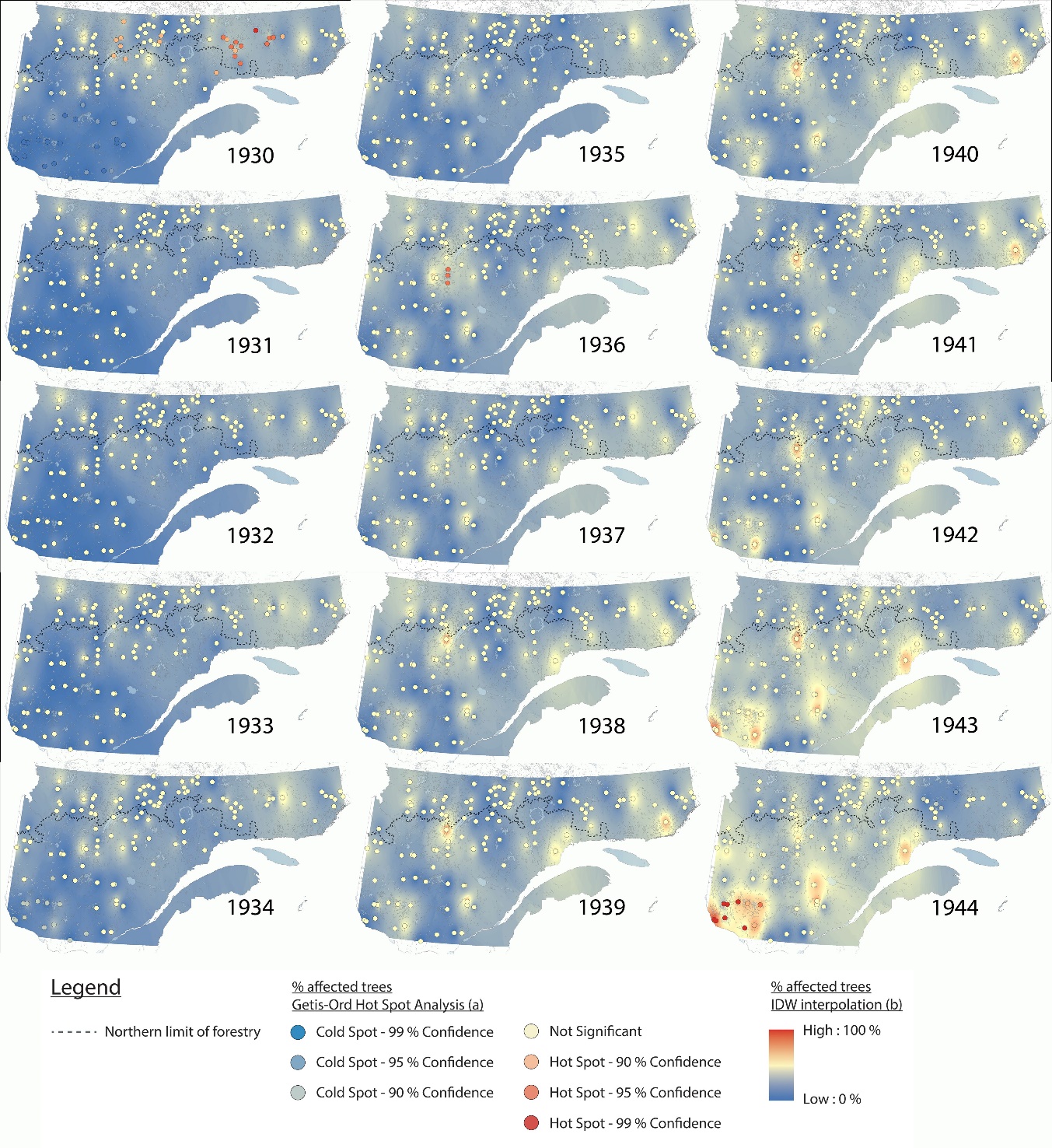


**Figure S1d.** Spatiotemporal pattern of spruce budworm impacts (percentage of affected trees) and synchrony (Getis-Ord hotspot analysis) in eastern Canadian boreal forests for the period 1944–1959.


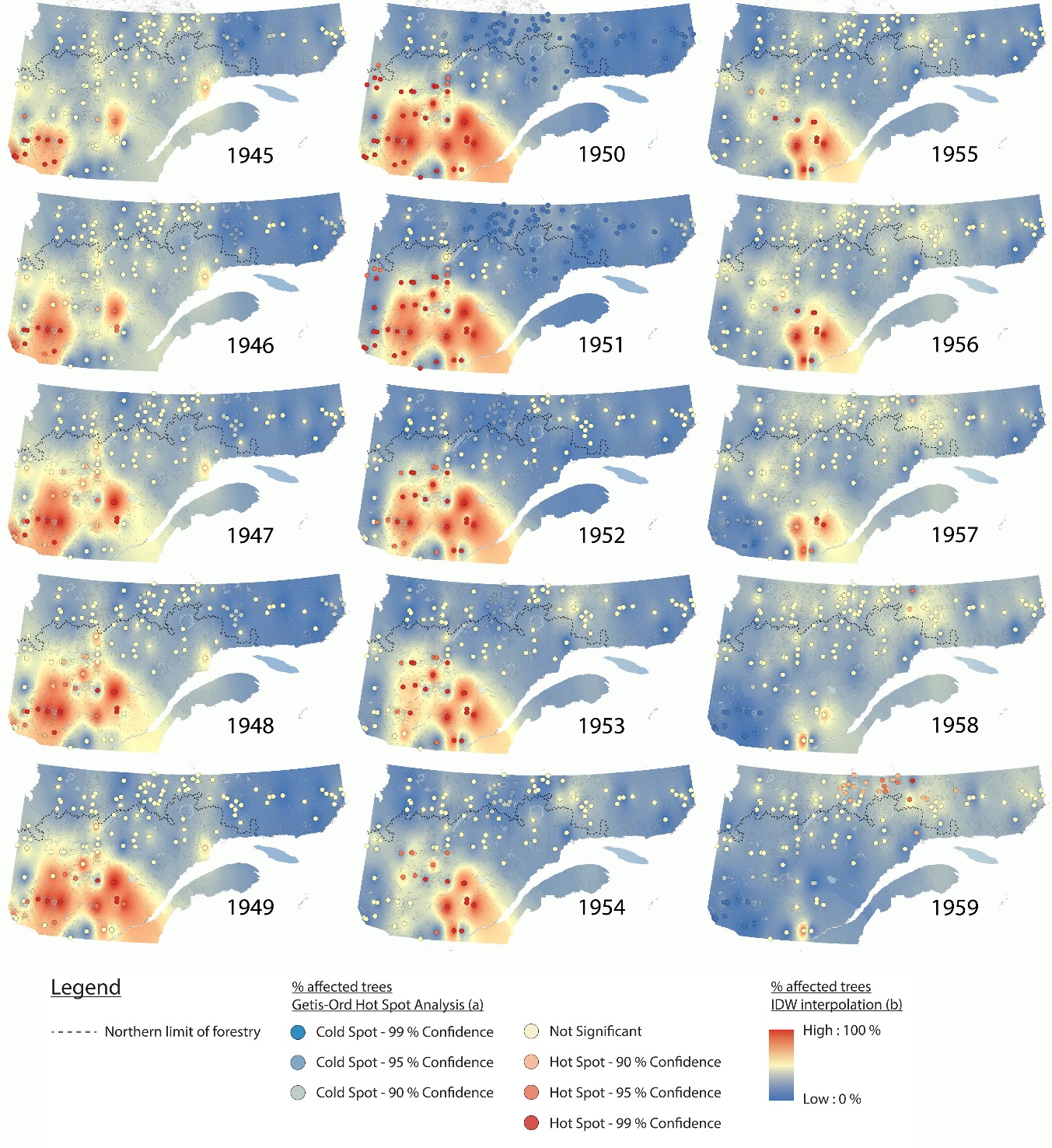


**Figure S1e.** Spatiotemporal pattern of spruce budworm impacts (percentage of affected trees) and synchrony (Getis-Ord hotspot analysis) in eastern Canadian boreal forests for the period 1960–1974.


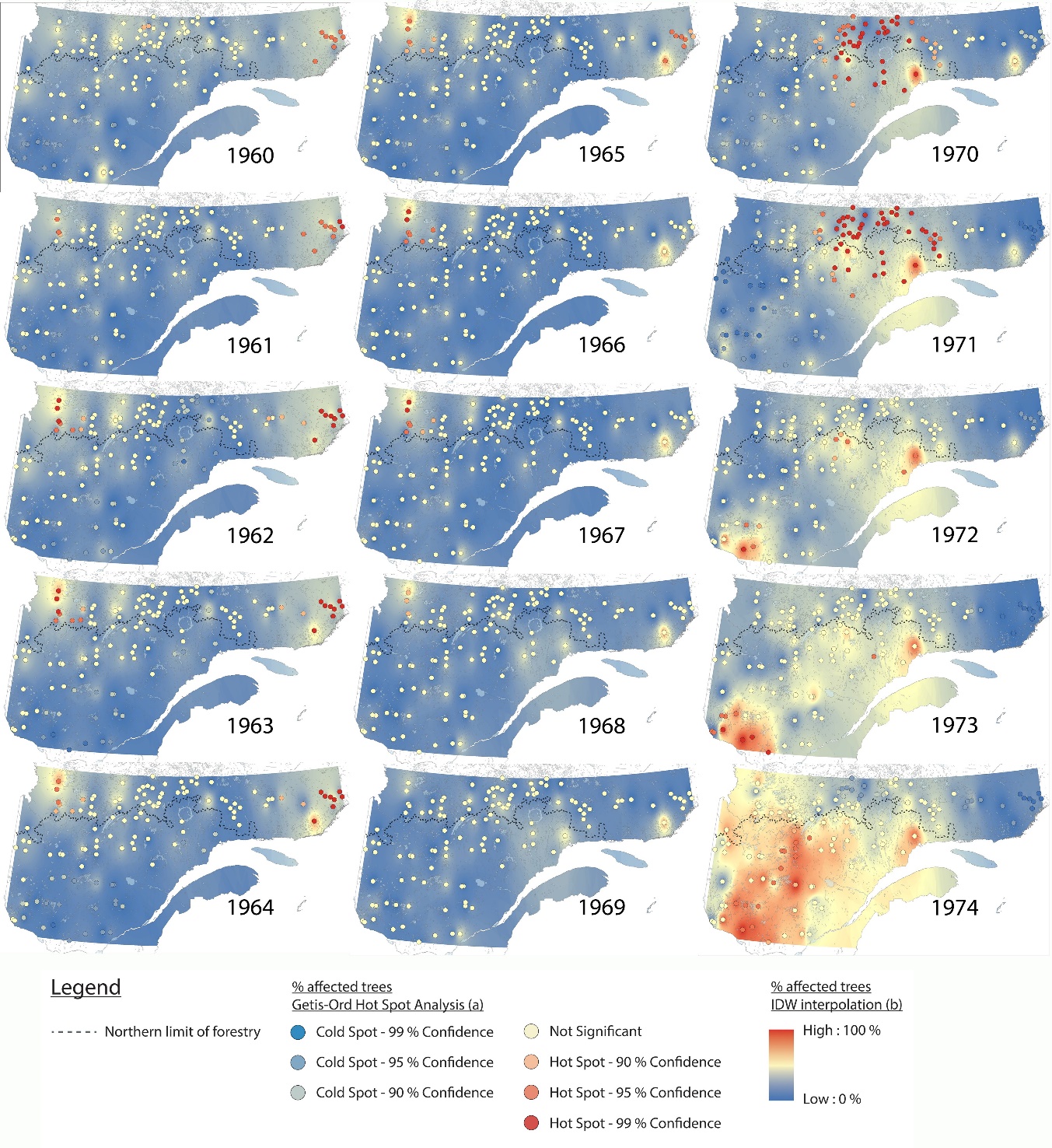


**Figure S1f.** Spatiotemporal pattern of spruce budworm impacts (percentage of affected trees) and synchrony (Getis-Ord hotspot analysis) in eastern Canadian boreal forests for the period 1975–1989.


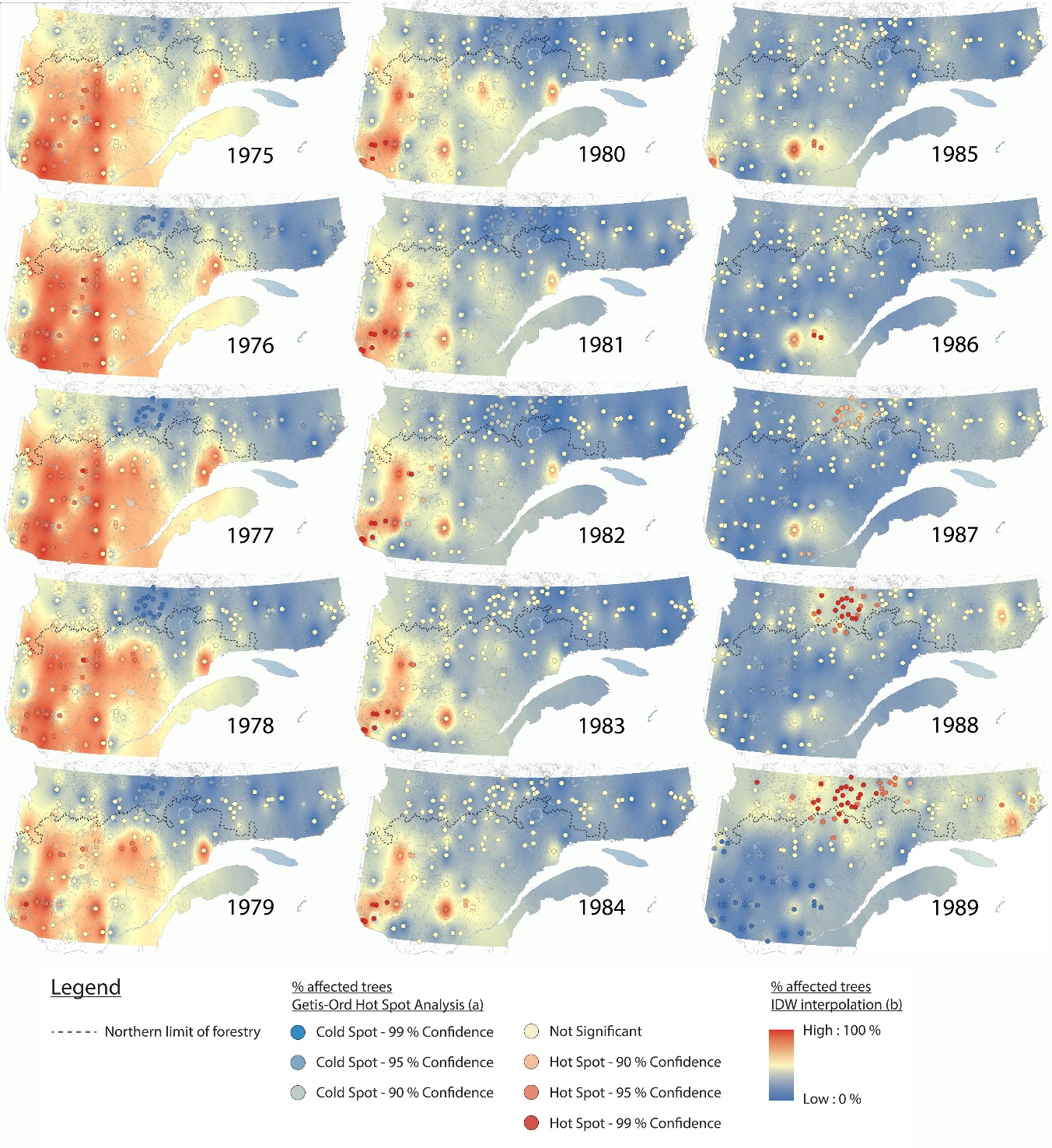

Supplement: Supplementary file 1 [file Table_1.docx]
